# Closed Loop Experiment Manager (CLEM)—An Open and Inexpensive Solution for Multichannel Electrophysiological Recordings and Closed Loop Experiments

**DOI:** 10.3389/fnins.2017.00579

**Published:** 2017-10-18

**Authors:** Hananel Hazan, Noam E. Ziv

**Affiliations:** ^1^Faculty of Medicine, Technion, Haifa, Israel; ^2^Network Biology Research Laboratories, Lorry Lokey Center for Life Sciences and Engineering, Technion, Haifa, Israel

**Keywords:** closed-loop system, electrophysiology, multielectrode array, software, network dynamics

## Abstract

There is growing need for multichannel electrophysiological systems that record from and interact with neuronal systems in near real-time. Such systems are needed, for example, for closed loop, multichannel electrophysiological/optogenetic experimentation *in vivo* and in a variety of other neuronal preparations, or for developing and testing neuro-prosthetic devices, to name a few. Furthermore, there is a need for such systems to be inexpensive, reliable, user friendly, easy to set-up, open and expandable, and possess long life cycles in face of rapidly changing computing environments. Finally, they should provide powerful, yet reasonably easy to implement facilities for developing closed-loop protocols for interacting with neuronal systems. Here, we survey commercial and open source systems that address these needs to varying degrees. We then present our own solution, which we refer to as Closed Loop Experiments Manager (CLEM). CLEM is an open source, soft real-time, Microsoft Windows desktop application that is based on a single generic personal computer (PC) and an inexpensive, general-purpose data acquisition board. CLEM provides a fully functional, user-friendly graphical interface, possesses facilities for recording, presenting and logging electrophysiological data from up to 64 analog channels, and facilities for controlling external devices, such as stimulators, through digital and analog interfaces. Importantly, it includes facilities for running closed-loop protocols written in any programming language that can generate dynamic link libraries (DLLs). We describe the application, its architecture and facilities. We then demonstrate, using networks of cortical neurons growing on multielectrode arrays (MEA) that despite its reliance on generic hardware, its performance is appropriate for flexible, closed-loop experimentation at the neuronal network level.

## Introduction

A common manner for studying complex dynamic systems is to use closed-loop systems that fix or control one or more interdependent variables; by uncoupling interdependent variables, such closed-loop systems allow these interdependencies to be resolved. The best-known example in the Neurosciences is the Voltage Clamp system, in which the control of membrane potential provides the means for resolving the dependence of ion-channel conductance on membrane potential. The use of closed-loop control systems, however, has expanded far beyond this purpose (reviewed in Wallach, 2013; Krook-Magnuson et al., 2015; Wright et al., 2016; see also Potter et al., 2014 and references therein). The advent of new stimulation and manipulation modalities (e.g., optogenetics) opens new and exciting possibilities for studying neuronal network functions or controlling network activity (e.g., Fong et al., 2015; Newman et al., 2015), increasing the need for fast, multichannel closed-loop control systems. Indeed, quite a few systems, based on different platforms, have been developed for this purpose. Preferably, such systems would be inexpensive, expandable, and customizable, easy to use, and provide user-friendly interfaces and development environments. Unfortunately, this is not always the case, and many of such systems represent tradeoffs between performance, complexity, ease of development, ease of use, expandability, dependence on specialized vs. common platforms, and of course, cost.

Given this state of things, we sought to develop a closed-loop system, which addresses some of the shortcomings mentioned above. Specifically, we developed an application for the widely used Microsoft Windows operating system, based on an inexpensive general purpose, 64-channel data acquisition board and a single, generic personal computer (PC). This application, which we refer to as Closed Loop Experiments Manager (CLEM), provides a full-featured graphics user interface (GUI) and possesses facilities for recording, displaying, logging, and playing back continuous electrophysiological data and/or individual action potentials from up to 64 analog channels, as well as facilities for reading from and activating external devices such as stimulators. Importantly, CLEM includes facilities for running closed-loop protocols that can be written in any programming language that can generate Microsoft Windows-compatible dynamic link libraries (DLLs), which are thereafter treated as “plugins.” The application which also includes several useful tools (such as an ability to run automated experiment sequences), does not depend on propriety libraries, is based on generic Microsoft Windows application programming interfaces (APIs), and is written in C and C++, with all source code available online.

We first survey current approaches and existing solutions, based on propriety platforms or open source code. We then describe our own solution and examine its performance using networks of cortical neurons growing on multielectrode array (MEA) dishes.

### Existing approaches and solutions

In general, closed-loop control systems face several rather challenging demands: Such systems are required to obtain data, process it, and generate output rapidly within short, well-defined durations. In addition, they are to store incoming data and control values to some permanent storage device. Preferably, they should also provide user feedback (render the data and display it) and remain responsive to user input. Several approaches have been taken to meet these requirements: (1) Systems built around real-time hardware; (2) hybrid systems composed of real-time hardware (which runs dedicated software) and non-real-time hardware, typically a general-purpose PC running mainstream software, or (3) systems built around a PC running real-time or soft real-time software.

Ultimately, the approach chosen depends on the particular requirements of such a system. Most important in this respect is the duration of each sample-analyze-output loop. Just as important is the ability to guarantee that these durations will be repeatable and that the system will not suffer from occasional, unacceptably long latencies. Clearly, the requirement for loop duration depends on the phenomena one wishes to study. For single channel biophysics, these durations must be on the order of microseconds; for network activity phenomena, however, which is the realm addressed here, sample-analyze-output loop durations matched to the duration of single action potentials (~1–2 ms) are probably acceptable. In any case, jitter in loop durations must be kept to a minimum and occasional longer latencies are to be avoided entirely.

Each one of the three approaches mentioned above is described next with the following considerations in mind: (1) real-time performance; (2) system complexity; (3) flexibility; (4) ease of software development (coding and debugging), and (5) cost and long-term maintenance. A summary covering some existing solutions is provided in Table 1.

**Table 1:** A comparison of several closed loop solutions (the list is not exhaustive).

**Program name**	**Operating system**	**Number of data acquisition cards**	**Analog I/O channels**	**Digital IO lines**	**Programming language (IDE)**	**Graphic user interface**	**Max sampling rate**	**Software extensions**	**Min. sample-analyze-output loop duration**	**Real time type**	**Type**
RTXI^a^	Linux + Xenomai extension	1 Supported by Analogy	2/2	None	C++ (GDB)	Yes	40 kHz	C++ Plugins	50 μs ± 1.5 μs	Hard	Generic PC
LCG^b^	Linux + Comedi extension	1 Supported by Comedi	2/2	None	C++, Python (GDB)	No		Python plugins		Hard	Generic PC
HyNNet^c^	Windows XP	Multiple specialized cards (32 for a full system)	4–60/4–30	64	C, C++	Yes	40 kHz	No	46 μs	Hard	Hybrid
CMOS-MEA, Neurotalker^d^	Linux	MEA-ASIC + FPGA	128/128		VHDL, C++		20 kHz	No	1.2 ms	Soft	Hybrid
Neuro-Righter^e^	Windows	2 National Instruments	64/4	32	C# (VS)	Yes	25 kHz	C# Plugins	StimSrv 46.9 ± 3.1 ms NewData 7.1 ± 1.5 ms	Soft	Generic PC
Matlab + Simulink^f^	Windows (host) QNX (target)	1 National Instruments	32/		Matlab + Simulink + RT-Lab	Yes	10 kHz	Matlab + Simulink	1 ms	Hard	Hybrid
Matlab + Simulink^g^	Windows (host) xPC (target)	1 UEI	64/2	16	Matlab + Simulink (Matlab)	Yes	46 kHz	Matlab + Simulink	1.5 ms ± 2.5 μs	Hard	Hybrid
Matlab + Simulink^h^	Windows (host) xPC (target)	1 National Instruments	32/4	48	Matlab + Simulink (Matlab)	Minimal	15 kHz	Matlab + Simulink		Hard	Hybrid
FPGA based system^i^	Linux	1 Virtex II pro	126/2		VHDL	No	20 kHz	No	FPGA: 400 ± 50 μs LAN: 83 ms ± 21 ms	Hard	SOC
LabVIEW Real Time^j^	Windows	4 National Instruments	64/2	16	LabVIEW Models (Labview)	Yes	100 kHz	LabVIEW Models	1 ms	Hard	Hybrid
Multi-Channel Systems^k^	Windows	Propriety electronics and onboard DSP	Up to 256 + 8/0	16	Visual dataflow programming (Experimenter)	Yes	50 kHz	.net McsUsbNet	1 ms	Hard	Hybrid
Tucker-Davis Technologies^l^	Windows	DSP-based large scale data acquisition systems	Up to 128/ Up to 128	24	Visual DSP programming (RPvdsEx) Visual dataflow programming (Synapse)	Yes	50 kHz	RPvdsEx (on DSP) ActiveX (on host)	<1 ms (on DSP) 5–50 ms (TDevAccX) 2–30 sec (TTankX)	Hard/Soft	Hybrid
CLEM	Windows	1 UEI	64/2	16	C, C++	Yes	46 kHz	DLL Plugins (C, C++, etc.)	1.4 ± 0.01 ms	Soft	Generic PC

a *Lin et al., 2010; Ortega et al., 2014; Patel et al., 2017*.

b *Linaro et al., 2014*.

c *Bontorin et al., 2007*.

d *Hafizovic et al., 2007*.

e *Newman et al., 2013. StimSrv is a double buffering system, which requires relatively long times between updates to NI D/A output buffer. While StimSrv is slow in comparison to the NewData and microcontroller options, it provides an interface that is easier to use and allows uninterrupted delivery of arbitrary complex signal outputs. NewData is an unbuffered method that can only respond by generating finite samples or periodic control signals*.

f *Novellino et al., 2007*.

g *Zrenner et al., 2010*.

h *Biró and Giugliano, 2015*.

i *Müller et al., 2013*.

j *Bryant and Gandhi, 2005. Channel counts and sampling frequencies relate to hardware used, not implementation. Most of the hardware mentioned is no longer available for purchase, but comparable items from the same vendor are available (PXI-1031 + PXI-6224 + PXIe-6535 + PXI-6602 + PXIe-8100)*.

k *MEA2100 + Multi Channel Experimenter, user manual; API for code extensions (McsUsbNet.dll) compatible with MCRack*.

l *RZ/RX series + Synapse software. RPvdsEx is a visual studio for real-time processor programming. TDevAccX and TTankX are ActiveX interfaces for closed loop interaction at different hardware levels. Latencies can be reduced by implementing additional hardware (Ethernet connection, a PO8e interface). Unprotected access via TDevAccX must be used carefully, as inappropriate use can crash the entire application*.

*Closed loop latencies were tested in our hands on RTXI and CLEM; the rest of the information is based on published papers and websites. GDB = GNU Project debugger, usually used through the command line. VS = Microsoft Visual Studio*.

### Real-time hardware

Fast, real-time hardware controllers are usually based on system on a chip (SOC), field-programmable gate array (FPGA) devices, or custom hardware. In these approaches, sampling, output and computation, including digital signal processing (DSP), pattern matching or user defined control algorithms, are executed directly on the hardware controller. While such approaches often guarantee fast, and reliable performance, they suffer from several shortcomings. First, both SOC and FPGA units often lack resources (e.g., processing power, memory, access to user interface devices) which are readily available on current day PCs, limiting the amount of data available at any given time to closed-loop algorithms and calling for careful, resource-conscious programming. Second, programming these units is often based on specialized languages and/or environments, which can have limited expressive power and take time to master (but see Müller et al., 2013 for one attempt to mitigate FPGA programming challenges). This is particularly true for FPGA units whose programming involves special languages (such as Verilog and associated languages) and compilers used to translate programs into logic gate instructions. In many cases, the translated logic gate map is larger than FPGA capacity, requiring division into smaller pieces, a non-trivial process, in particular in programming environments that lack the modern debugging features of contemporary integrated development environments (IDEs). Third, while SOC units have sufficient computational power to perform the tasks they were designed for (e.g., DSP), they are often insufficiently powerful to perform general purpose tasks. In theory, multiple SOC boards can be used, but task division and coordination poses significant challenges, in particular to non-experts. Finally, when particular SOCs and FPGAs become obsolete, porting the code to newer units can prove to be a very laborious task. Given the enormous growth of PC computational capacities, the justification for this class of solutions is diminishing rapidly.

### Hybrid real-time and general-purpose systems

Given the limitations of dedicated hardware systems, hybrid solutions are commonly preferred. Here, tasks are divided between two subsystems: (1) A real time subsystem that handles time-sensitive, computationally-intensive tasks; this subsystem (often referred to as the “Target”) is typically a SOC, a FPGA or a dedicated stripped-down PC, which runs a (minimal) real-time operating system (OS) and contains data acquisition hardware. (2) A host system (typically a general purpose PC) that handles tasks that are less time-sensitive such as display, user interface, and data logging. The two subsystems are connected internally through standard buses (e.g., PCI) or externally through communication interfaces such as USB or Ethernet. During execution, binary files, created on the host, are downloaded to the real-time system and executed, with the aforementioned channels serving to transfer data, commands, and alerts between the two subsystems.

As the hybrid approach combines the advantages of each subsystem while overcoming many of their limitations, it is generally viewed as attractive solution. In the neuroscience community, this hybrid approach has gained popularity due to the availability of a Matlab compatible solution, namely Simulink Real-Time, in which the target is a dedicated stripped-down PC (xPC Target). Such hybrid systems were used successfully to “embody” *in-vitro* networks in physical “robots” (Cozzi et al., 2005; Novellino et al., 2007), to study and clamp neuronal response probabilities to electrical stimuli and synaptic input (Wallach et al., 2011; Wallach and Marom, 2012; Reinartz et al., 2014) and to clamp network synchrony levels through timed neuromodulator delivery (Kaufman et al., 2014). In these studies, the host computer ran Matlab scripts within the Matlab environment whereas the software running on the target was programmed using Simulink, a graphical programming environment based on blocks and connections. This architecture highlights the fact that hybrid systems typically call for the development of two separate applications, one that runs on the host, and one that runs on the target. In practice, development of these applications often differs substantially, requiring the experimenter or developer to master two different programming languages and their respective development environments.

Another useful commercial solution is LabVIEW Real Time (e.g., Bryant and Gandhi, 2005). Software development within this framework is based on an elegant and powerful graphical programming interface which, from the developer/user's standpoint, does not differ substantially for applications running on real time targets or the host. This unified “point and click” interface greatly simplifies development, integration, and testing of closed loop systems, while hiding many tedious low level implementation details. A large number of SOC, FPGA, and DSP targets are compatible with this framework, providing modular and extendable architectures. It should be noted, however that these systems tend to be quite costly. It should also be mentioned that using the elegant graphical programming interface still requires a good grasp of signal processing, timing, and closed-loop programming concepts. Consequently considerable time investment is still needed, in particular for experimentalists without suitable backgrounds.

Commercial solutions exist from additional vendors such as Multichannel Systems (MCS) and Tucker-Davis Technologies. These vendors offer comprehensive hardware and software suites suitable for a wide range of experimental needs, which include powerful graphical programming interfaces for controlling data acquisition, processing, display, logging, and analysis. In addition, these suites provide close-loop functionality based on dedicated hardware and standardized sets of conditions, For example, the Real Time Feedback option in the “Multi Channel Experimenter” application (MCS) allows for closed-loop stimuli generation based on detection of spikes (or predetermined numbers of spikes) in certain recording channels within predefined time windows. Such conditions are typically tested on the real-time subsystem, resulting in very short latencies (a few ms).

Although hybrid systems are both advantageous and useful, they too have certain shortcomings. First, they require two separate subsystems (e.g., a generic PC and a target) increasing costs and overall complexity. Even when the target is an inexpensive, stripped down PC (a common choice), its configuration (e.g., BIOS settings) needs to be fine-tuned to assure good performance and avoid sporadic latencies. Other targets for Simulink and LabVIEW Real Time exist, but these are usually much more costly. Second, the use of two subsystems implies two separate applications as discussed above, with the development complexities this entails. While such complexities can be hidden from the experimenter through user-friendly graphical programming environments, these do not cater to every scheme, creating difficulties when the loop is to be closed on complex features of incoming data, for which no boilerplate function or graphical object exists. For such non-standard schemes, some commercial systems provide APIs that allow experimenters to write their own code using general purpose programming languages (e.g., C, C++). For instance, Tucker-Davis Technologies offers APIs that allow programs written in a variety of languages (including Matlab) to access their core modules and data repositories through ActiveX interfaces. It should be noted, however, that this complex software layer can introduce significant delays and impair robustness (see Table 1 for further details). Thus, for demanding experiments, the complexities of hybrid systems are difficult to evade, and the use of flexible and expressive general purpose programming languages becomes unavoidable.

### Real-time or soft real-time software controllers

The dramatic increases in computational power and the prevalence of multicore processors have led to an emergence of new and inexpensive solutions based on generic PCs. Here, good to excellent hard or soft real-time performance can be attained, depending on the OS installed and closed-loop timing requirements. The advantage of this approach is found in its low cost and simplicity. Furthermore, a huge variety of excellent, powerful and user-friendly programming environments is available for these platforms, facilitating system and protocol development. The main disadvantage of this approach concerns the fact that mainstream OSs are not optimized for real time performance. This matter deserves some elaboration.

OSs provide high-level interfaces through which running applications access computer resources (memory, file system, network, display, user interface devices, etc.). Among others, OSs time-share application execution through a scheduling mechanism, providing the illusion that multiple applications and background services run concurrently, even though at any given time only a small number of tasks is actually executed. General-purpose OSs such as Microsoft Windows, MacOS, and Linux employ dynamic scheduling systems that prioritize tasks, dividing execution time between applications according to their priority, providing the user with a responsive experience. Inessential programs and high priority tasks, however, can slow system responsiveness and introduce delays. Thus, scheduler behavior in such OSs limits their ability to serve as real-time systems.

Contrary to general-purpose OSs, real-time OSs aim to guarantee soft or hard execution deadlines, at the expense of general performance. The schedulers' behavior determines if the OS is a hard real-time or soft real-time system: If deadlines are usually met, the system is considered a soft real-time OS; if deadlines are met deterministically, the system is considered a hard real-time OS. Thus, execution will be most precise (i.e., execution loop durations will show least jitter) on hard real-time OSs, slightly less precise on soft real-time systems and least precise on general-purpose OSs. Some examples of real time OSs include Xenomai (https://xenomai.org/) and RTXI (http://rtxi.org/)—open source, real-time Linux frameworks and RTX/RTX64—a propriety, real-time Windows framework (https://www.intervalzero.com/).

Another consideration is minimal execution time slice duration. Thus, for example, Microsoft Windows typically achieves a granularity of about 1 ms. By contrast, Xenomai provides a minimum granularity of 59 μs (Brown and Martin, 2010) whereas RTXI provides a granularity as low as 50 μs (Patel et al., 2017). Along these lines, Linux has been quite widely adopted by the scientific community and has growing user and support bases, due to its open source and free of charge policy, and, importantly, the freedom to change any part of its operation. Yet, Linux has rightfully earned a reputation of having a steep learning curve, and its development environments are not as user friendly or diverse as those available for mainstream OSs. Moreover, Xenomai is not compatible with all Linux distributions and versions, and requires significant knowledge to set up and maintain. This situation is generally true for other Linux based solutions, such as RTXI (Lin et al., 2010; Ortega et al., 2014; Patel et al., 2017) and LCG (Linaro et al., 2014). Commercial real-time systems have better and more centralized support and migration paths, but these systems can be expensive and rely on code that is hidden from the experimenter. Therefore, it would be desirable that single PC systems would run over mainstream OSs, which typically have streamlined migration paths and offer significant backward compatibility.

### Selected solution-overview

Our aim was to develop a simple and reliable system that would allow for closed-loop experiments with networks of spiking neurons. Placing the pros and cons of the three approaches listed above against this aim led us to favor the third approach. Furthermore, as we aimed for a user-friendly system with a reasonably long life cycle, we elected to develop our system over Microsoft Windows, minimizing dependence on unique products with uncertain futures, such as niche OSs and third-party software libraries. Given that the duration of a single action potential is ~1–2 ms, the minimal granularity of 1 ms of this OSs' scheduler was deemed sufficient. The system was thus built around a single generic PC running Microsoft Windows and a single general purpose, PCI data acquisition board (PD2-MF-64-3M/12, United Electronics Industries; Table 2). A similar approach - the NeuroRighter Electrophysiology Platform - has been recently described (Rolston et al., 2010; Newman et al., 2013; Laxpati et al., 2015). This platform has been used successfully to control firing in neuronal populations using optical methods (Newman et al., 2015) and derive the roles of spiking and neurotransmission in driving upward scaling of synaptic strengths (Fong et al., 2015). In this platform, however, closed loop latencies, were slightly excessive (~7 or 49 ms; depending on the closed-loop mode). Furthermore, two, 32 channel data acquisition boards were used, increasing system cost and complexity. In contrast, the system described here is based on a single data acquisition board that can amplify and sample analog data from 64 input channels at sufficiently high rates (>45 kSsamples/s), provide concomitant analog (2 channels) and digital output (32 channels) without requirements for additional external devices (e.g., multiplexers, amplifiers etc.). Consequently, the cost of the entire system (data acquisition board, PC, monitor, software) is <$3,000.

**Table 2:** CLEM components and costs.

**Item**	**Rig 1**	**Rig 2**	**Cost ($US)**
Brand	Dell optiplex 9010	Generic	600
Motherboard	Dell	Intel DB75EN	
CPU	i7-3770 @ 3.4 Ghz	i7-3770 @ 3.4 Ghz	
RAM	8 Gbyte (DDR3)	4 Gbyte (DDR3)	
Data acquisition card	UEI Power DAQ PD2-MF-64-3 M/12 H	UEI powerDAQ PD2-MF-64-3 M/12 L	2,200
Operating system	Windows 7 enterprise	Windows 7 enterprise	Free for academic use^*^
Programming environment	Visual studio 2013 (C and C++)	Visual studio 2013 (C and C++)	

*Two slightly different rigs were built and are currently in routine use*.

* *Within Microsoft's restrictions*.

As described above, Microsoft Windows is not a real-time OS; most contemporary data acquisition boards, however, provide asynchronous acquisition modes in which the board transfers incoming data into PC memory autonomously. Typically, interrupts are generated each time the transfer of predetermined numbers of samples is completed. These events can be used to clock sample-analyze-output loops. We found that this mode is sufficiently reliable to support closed loop performance with latencies under 2 ms with practically no jitter (see below). In addition to this hardware-timed mode, we implemented a slower mode, detached from hardware timing mechanisms, in which sample-analyze-output loop duration is user defined, for (concomitant, if so desired) use with tasks that are less time sensitive and/or require lengthy computations.

**Figure 1:**
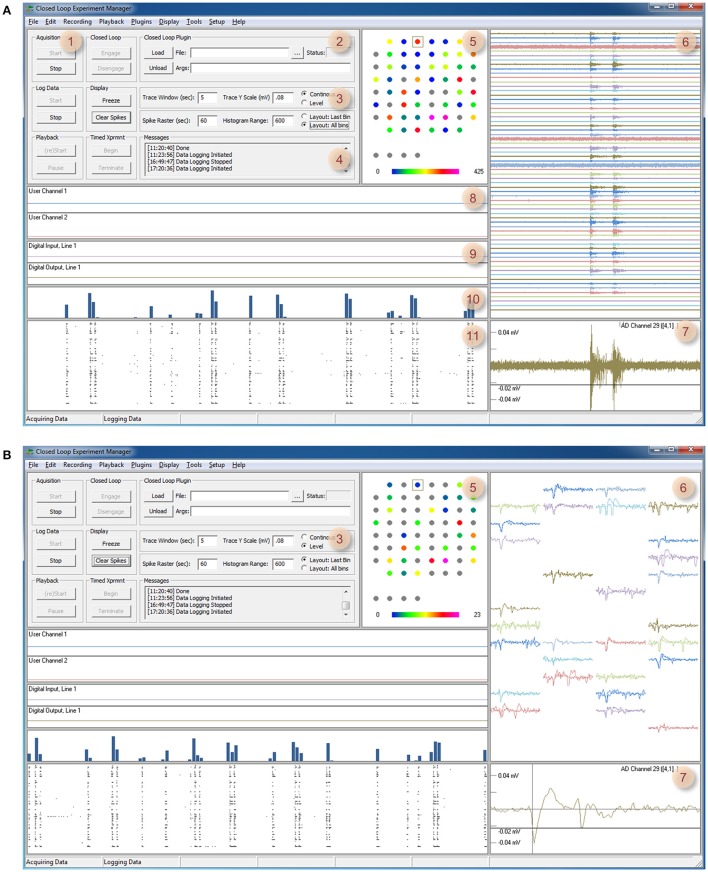
CLEM's Graphical user interface. **(A)** A screenshot of CLEMs main window (Continuous mode). The left top region contains readily accessible controls, whereas all other regions display incoming data and data statistics. (1) Controls for starting and stopping data acquisition, data logging, playback, and timed experiments. (2) Controls for loading and engaging closed loop “plugins.” (3) Controls for changing display modes and settings. (4) Text messages generated by CLEM and closed-loop functions. Double clicking on this window allows manual entry of *ad-hoc* comments. (5) Channel physical layout. Custom layouts can be created using any text editor and thereafter loaded from the Options menu. Cumulative spike rates are color coded according to color bar below. (6) Continuous voltage recordings from all channels. The time window and vertical scale are defined in region 3. (7) Magnified view of one channel. The particular channel is selected by double clicking on one of the traces or channels in regions 5 or 6. The threshold used for spike detection (black horizontal line) can be moved up and down interactively. (8) User data display—two channels of data generated by plugin functions can be displayed here. (9) Digital input and output, one line for each plot. IO lines to be displayed can be selected from the menu. (10) Histograms of spikes detected in all channels. (11) Rasters of spikes detected in all channels. The time window of regions 8–11 is defined using the controls in region 3. **(B)** A screenshot of the main window in Level mode. Here (6,7) waveforms of the most recent spikes detected in each channel (up to 10 most recent spikes) are displayed instead of continuous voltage values. Note also that region 5 shows momentary, rather than cumulative spike numbers (display mode controlled in region 3).

**Figure 2:**
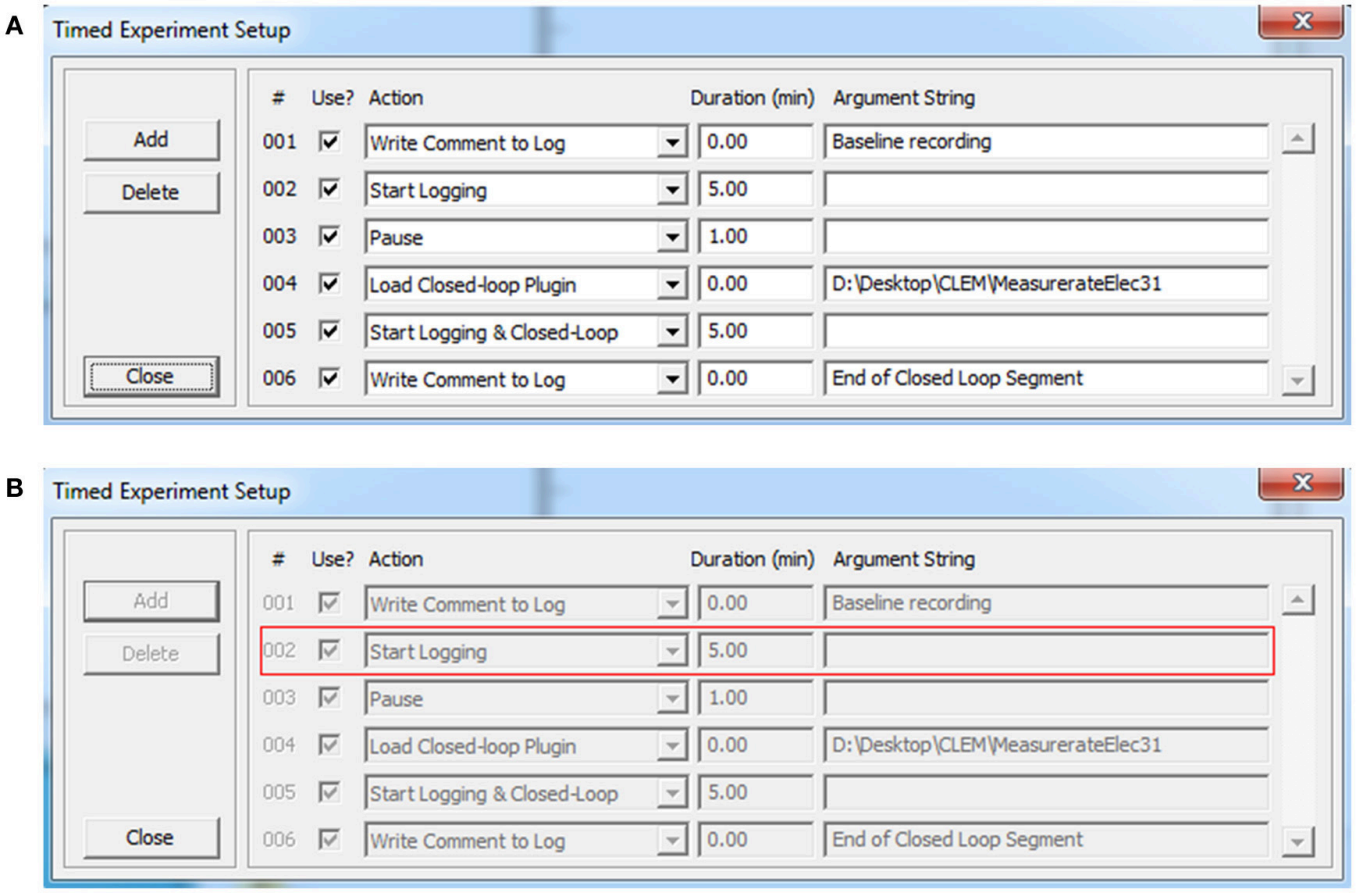
Timed experiments. **(A)** Automated execution of arbitrary experiment sequences can be carried out programmatically using this dialog box and adding particular actions and their respective durations (up to 200 actions). Actions are selected out of a predefined list that includes, among others, loading plugins, and executing the functions they contain. Arguments can be provided as needed. Experiment sequences can be stored to file and thereafter reused. **(B)** When the experiment sequence is initiated (Figure 1A, region 1) CLEM steps through the actions and executes them sequentially. The software highlights the action being executed with a red rectangle.

In addition to closed loop tasks, the system stores and displays incoming and outgoing data, detects and displays spikes, displays spike rasters and histograms, activity statistics for spatially rendered channels, digital and analog output and data generated by closed loop routines (user data). Furthermore, the system displays and stores (to the desktop, by default) a text-based log of all activities including comments entered by the experimenter or generated by closed-loop procedures. Finally the system has facilities for performing timed experiment sequences, has provisions for playing back stored data, and several additional tools. An overview of the graphical user interface is provided in Figures 1, 2.

### Software architecture

**Figure 3:**
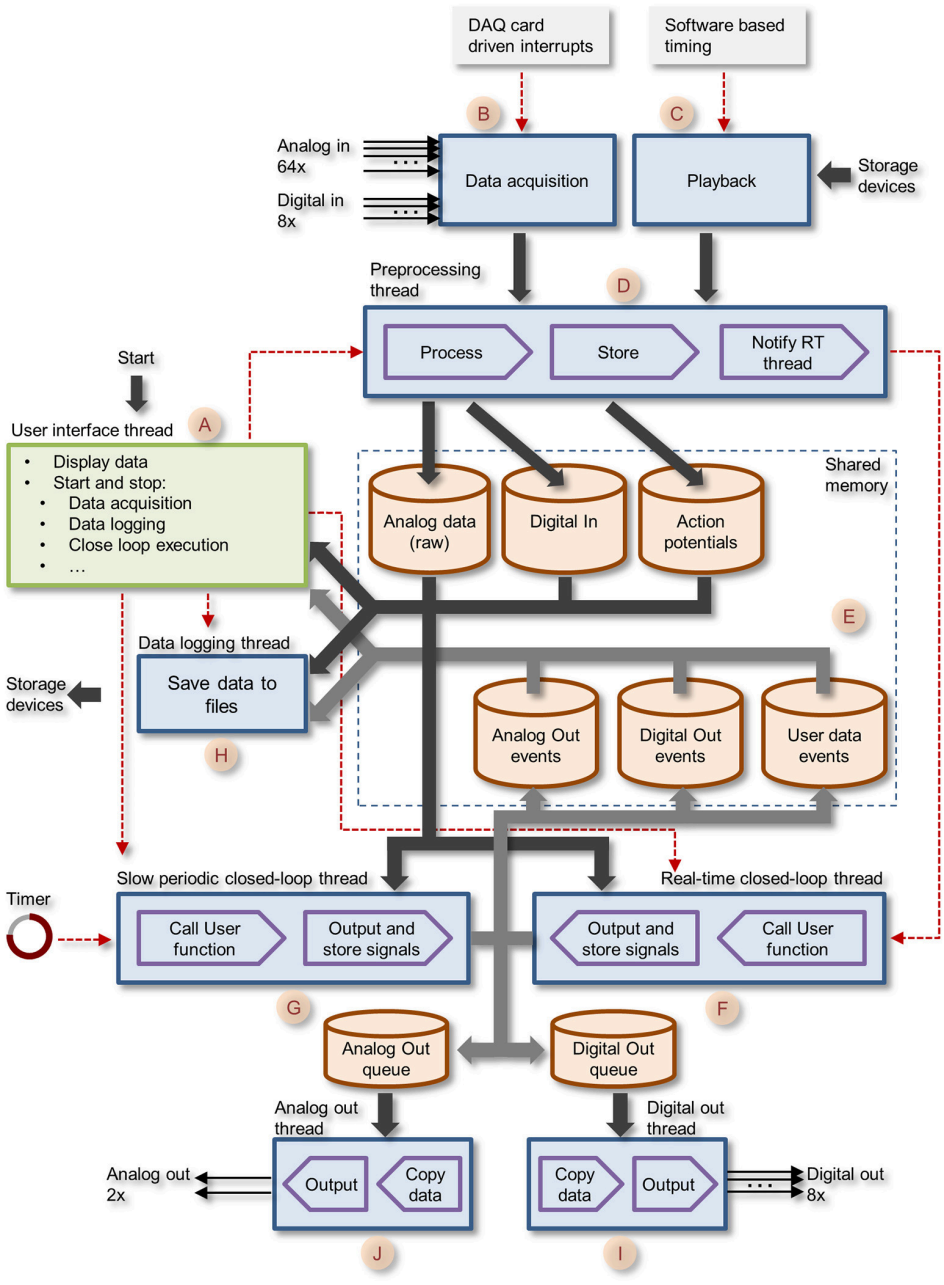
Block diagram of CLEM showing the different threads and central memory repositories. The GUI thread is shown in green, all core function threads are shown in blue. Data paths are shown as thick arrows. Control lines are shown as red dashed lines.

The entire application was written as a Windows desktop application in C and C++, and is based on generic APIs, with practically no dependence on external software libraries beyond those provided by the data acquisition board vendor (Table 2). The overall architecture of the system is illustrated in Figure 3. The application has two main thread groups: (1) the GUI thread, and (2) the Core Functions threads. Threads run in parallel, with communication between threads realized through shared memory. To take advantage of performance improvements provided by contemporary multicore processors, Core function tasks are divided between multiple threads.

Data collected is stored in memory repositories, which are shared by and accessible to all running threads. These include raw voltage traces of all 64 analog channels; Action potential (spike) events; Digital In events; Digital Out events; Analog Out events, and User data events (i.e., data generated by closed loop routines as explained later). These repositories contain the most recent data, with predefined history durations. As the main aim was to interface with spiking networks, data repositories were designed to store the histories of several hundreds of thousands of action potentials (time, channel/electrode, waveform). Apart from raw voltage data, which is stored as a continuous stream of numerical values, all other data forms are stored as time-stamped events, i.e., an entry containing a time stamp (in sample units) and data specific to each event type. For spikes, each event contains, in addition to the time stamp, the particular channel on which it was detected and a waveform of the voltage trace of the particular spike. Spikes are detected as threshold crossing events and the waveform contains data within a user defined time window covering the period just before and after the threshold-crossing event. Unlike other systems, multiple spikes occurring within one such time window are not lumped into a single event but are stored as separate (overlapping) events.

User defined closed-loop programs are treated as “plugins” that can be written in any programming language that can produce a standard Window dynamic link library (DLL). These DLLs must conform to certain specifications as described below. DLLs are loaded, activated, stopped and unloaded dynamically by the user. When running, plugin routines have full access to all data stored in central repositories, can use these data to calculate actions (or lack thereof) and then generate output requests, which are thereafter handled by suitable core function threads. User-defined closed-loop routines are developed independently from the main application, allowing the experimenter to write, load, debug, and test these without leaving the main application. These plugins can also generate and store, if so desired, two streams of analog values (User data events) which are displayed on the GUI, representing, for example, control values related to closed-loop procedures (e.g., stimulation intensity) or values calculated on the fly (e.g., spike rates, measures of synchrony, etc.).

All source code, as well as installation files for an executable version are available at https://github.com/Hananel-Hazan/CLEM

As mentioned above, execution is carried out in multiple threads that run concurrently. Below is a brief description of these threads.

### Graphic user interface thread

The graphic user interface thread handles all user interface using standard Windows controls and menus [Figure 3 (A)]. It then notifies core function threads [Figure 3 (B–J)] of user driven events such as requests to begin or stop acquisition, data logging, closed loop execution, playback, etc. The graphic user interface also displays the most recent data residing in data repositories, basic information on activity statistics and spatial sources (using user-defined source layouts) as illustrated in Figure 1. It also provides facilities for loading, engaging, disengaging and unloading closed-loop plugins. Finally, it provides facilities to carry out predefined, timed experiment protocols as illustrated Figure 2. Here, the user can create or edit a list of tasks (up to 200). Each task is selected from a predefined list that includes, among others, data logging, loading plugins and executing the functions they contain. The duration of each task is defined and arguments can be provided as needed. Task sequences can be stored to files and thereafter loaded and reused. When the experiment sequence is initiated through the user interface (Figure 1A, region 1) CLEM steps through the actions and executes them sequentially (Figure 2B), until the sequence is completed or terminated manually.

### Core function threads

The Core function creates eight separate threads that run concurrently as described schematically in Figure 3 (letters in parenthesis refer to annotations in Figure 3). These include: (1) the preprocessing thread (D)—handles blocks of incoming samples digitized by the data acquisition board, preprocesses the data, detects action potentials, and stores the data to central repositories; (2) the real-time closed-loop thread (F)—executes user defined, closed-loop procedures. Called each time the preprocessing thread completes the handling of a new data block; (3) The slow periodic closed-loop thread (G)—executes closed loop procedures at user defined time intervals; (4) The Analog Out thread (J)—outputs user-program-defined analog signals via data acquisition board digital to analog channels; (5) The Digital Out thread (I)—outputs user-program-defined digital signals via data acquisition board digital IO ports; (6) the Playback thread (C)—loads prerecorded data from storage devices and “injects” it into the incoming data pipeline (emulating the data acquisition process); (7) Data logging thread (H)—stores data to permanent storage devices.

**Figure 4:**
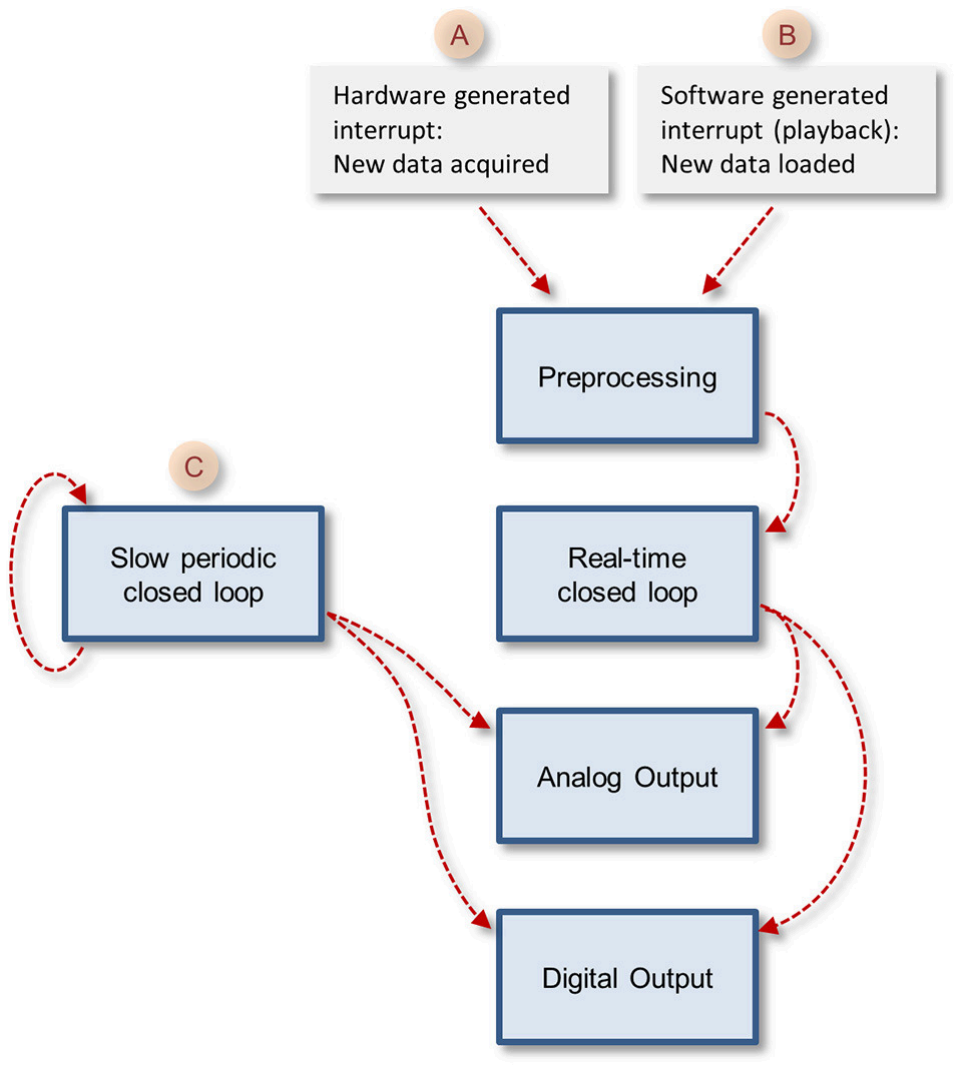
The closed loop pipelines. Note that the execution of each stage is timed by the previous stage in the pipeline, but each of these tasks runs in its own thread. The exception is the slow periodic closed loop pipeline that times itself based on a user-defined time interval.

The core function queue (Figure 4) operates in a pipeline fashion. The queue is initiated by either one of two interrupts: (1) a hardware interrupt [Figure 4(A)] initiated by the data acquisition board every time it completes the acquisition and transfer of a block of data samples to PC memory. (2) An emulated interrupt [Figure 4 (B)] initiated periodically during data playback. In the latter case, the playback procedure loads an equivalent block of samples from a storage device into PC memory, and then activates the preprocessing thread as would happen during data acquisition. Either interrupt starts the preprocessing thread, which processes incoming data, detects and stores spikes and places all new data in the main data repository [Figure 3 (E)]. It then notifies the real-time closed-loop thread that new data has arrived, and goes dormant, until evoked by the next interrupt.

When the real-time closed-loop thread is cued, it calls the user-defined closed loop procedure. It then checks if this procedure created output to be sent to the hardware output channels. If it did, the thread transfers the output to memory blocks it shares with the analog or digital output threads [Figure 3 (I,J)], notifies them that data is ready for output and then goes dormant.

When the analog and/or digital output threads are cued, they copy the data from the shared memory block to their internal memory and send it to appropriate channels on the data acquisition board; they then go dormant until cued again.

In addition to the hardware-clocked, real-time closed-loop thread, the system also runs a slow periodic closed-loop thread [Figure 4 (C)] which is called at user-defined time intervals. The use of these threads is not mutually exclusive, and both can be used concurrently, if so desired. Here too, after the user-defined closed loop procedure is completed, the thread checks if the procedure created output; if so, the thread transfers the output to shared memory blocks [Figure 3 (I,J)], notifies the output threads that data is ready and goes dormant. To avoid the possibility that both the real-time and slow periodic closed-loop threads write to the shared output buffer simultaneously, the buffer is protected (with a semaphore); note that as a result, attempts to write to this buffer from the slow periodic thread while the buffer is in use by the real-time thread might lead to loss of output from the slow periodic thread.

The data logging thread [Figure 3 (H)] runs every several of hundreds of milliseconds, and stores all data collected since its last iteration to a permanent storage device (e.g., hard disk). Data is stored to a series of files opened and closed sequentially and automatically such that files do not exceed a user-defined size. Data streams are stored separately for each data type: Analog (raw) input, spike events, digital IO events, analog out events, user-defined data events, and text messages generated by the system or entered by the user. Any or all data streams can be stored, according to user preferences. Data is stored using CLEM file formats, but a tool for converting Analog (raw) input and spike events to textual formats (for import into data analysis applications, for example) is included and accessible from the GUI menu. Conversion is carried out in a separate thread and thus can be performed concurrently with experiments.

### User-defined closed loop procedures

**Figure 5:**
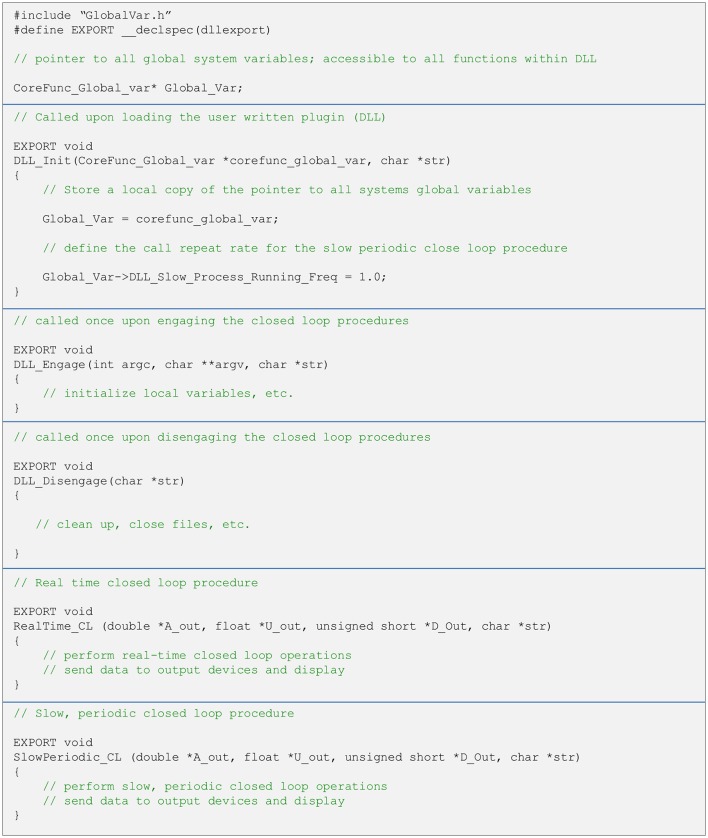
General organization of a plugin source file. The file should contain five functions with the exact signatures (i.e., arguments and data types) shown here (using C/C++ notation). These functions are loaded into memory when the plugin is loaded and called as explained in the comments (green text). The functions to do not return values and all data is passed back to CLEM through pointers. Strings are passed as pointers to NULL terminated arrays of 8 bit characters. The definition of the data structure “CoreFunc_Global_var” is provided in a separate header file (GlobalVar.h). For further details please see Supplementary Data.

As explained above, user written closed-loop procedures can be called in one of two fashions—as part of the real-time pipeline or using the slow periodic closed-loop thread. Both are called from a single DLL (note that it is not mandatory to implement both procedures). Regardless of how they are run, user written closed-loop procedures have the same format. Both functions receive pointers to central data repository items as well as additional variables. It is up to the user to avoid corrupting data in global repositories. The typical structure of a DLL source file, with placeholders and templates for the two types of close-loop procedures is illustrated in Figure 5. To maximize interoperability with DLLs written in other languages, arguments passed to the functions are based on generic data types. Source and header files, as well as detailed explanations for creating such DLLs in both C and C++ are provided as Supplementary Data, demonstrating the relative simplicity of implementing closed-loop routines using CLEM. Source files can also be found at https://github.com/Hananel-Hazan/CLEM-Plugin-Template.

When the user selects and loads such a DLL file, the core function loads the closed loop procedures into memory and incorporates them into the closed-loop pipelines (Figure 4). Thus, no explicit user-written actions are needed to call these procedures. The user, however, cannot change the names or signatures of these functions.

As mentioned above, the closed loop procedures can generate output for hardware lines (namely Analog and Digital output lines). In addition, they can display two continuous values they compute or output (for example, spike rate or stimulus amplitude), by sending data to the User data streams. These data streams are thereafter displayed as User data graphs [Figure 1 (8)] and can be streamed to a storage device if so desired. Finally, the procedures can generate text messages that are sent to the appropriate text message window in the GUI [Figure 1 (4)].

### Demonstration of closed-loop execution

As a demonstration of closed-loop procedure execution, we wrote a DLL which carries out two concomitant tasks. The first, carried out by the real-time closed-loop procedure, scans recent spikes for a predefined motif (see below); upon its detection, it triggers an external device through one of the digital-out lines. The second, carried out by the slow periodic procedure (called once a second), counts the number of spikes detected during the last 60 s and updates the User data graph accordingly.

To this end, CLEM was connected to a 60-channel MEA amplifier as described in section Materials and Methods. Networks of cortical neurons (~3 weeks in culture) growing on thin glass MEA dishes were mounted in the amplifier and recording was carried out to identify active electrodes. Motifs were then defined as sequences of action potentials recorded from five different active electrodes within inter-spike intervals that did not exceed 10 ms. The task assigned to the real-time closed loop procedure was to (1) scan the spike history repository in search of the predefined motif; (2) upon identifying one, set a digital out line to high; (3) set the digital line back to low after 5 ms, and (4) impose a refractory period such that no triggers will be issued within 1 sec of the prior signaled event, even if motif recurrences are detected. The task assigned to the slow periodic closed loop procedure was to (1) scan the spike history repository and count all spikes recorded within the last 1 min; (2) scale the value; and (3) send the value to the first user data graph. All code was written in C, compiled and tested by loading (and unloading) the DLL into CLEM without leaving CLEM.

**Figure 6:**
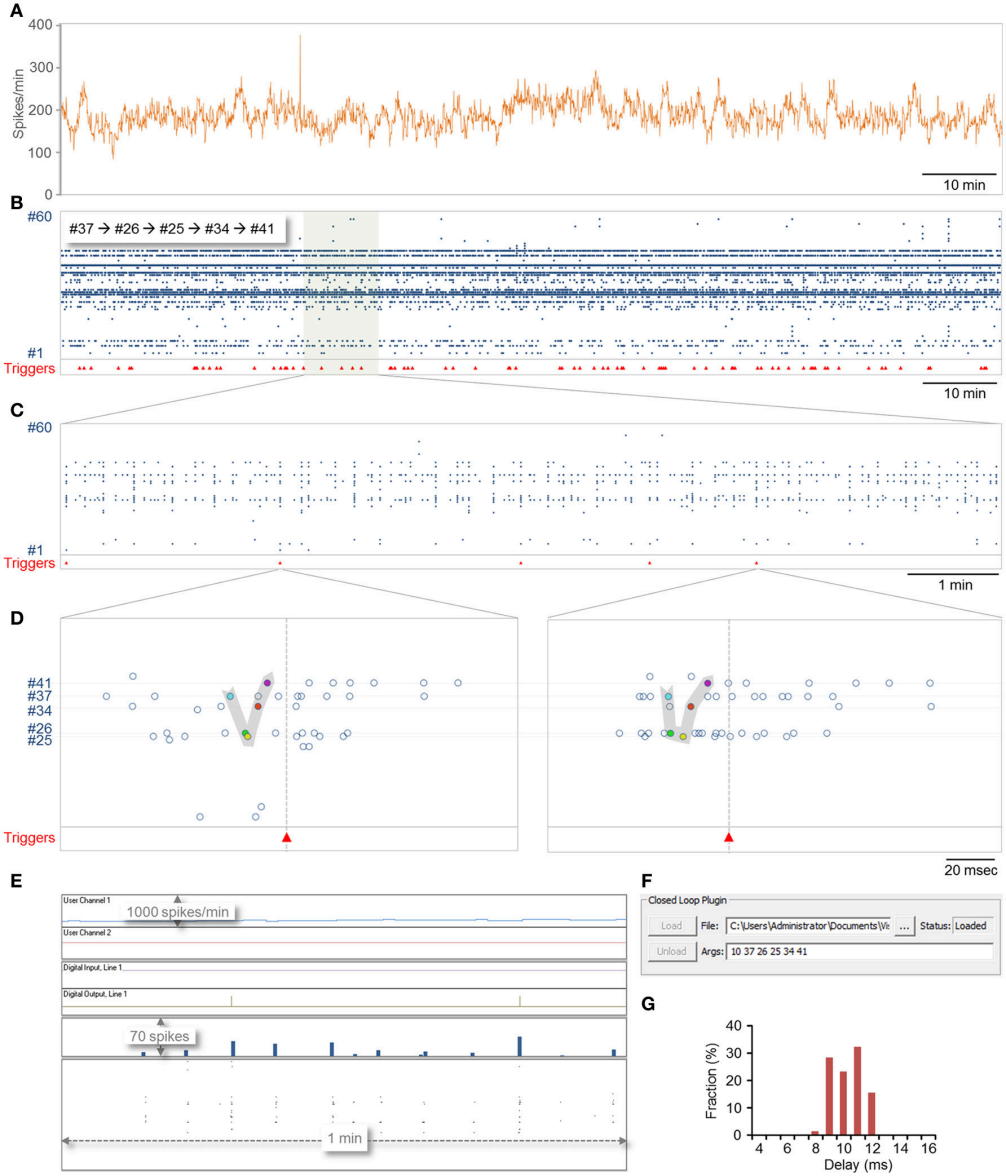
Demonstration of closed-loop plugin execution. CLEM was connected to a MEA amplifier recording from a network of cortical neurons plated on a 59 electrode MEA dish. A plugin containing both real-time and slow periodic closed-loop procedures was loaded, and run for 124 min. Sampling was carried out at 16 kHz per channel. **(A)** Spike rate, expressed in spikes per minute, calculated and logged on the fly by the slow-periodic closed loop procedure. This procedure was programmed to scan the spike history repository, count all spikes recorded from all electrodes within the last 1 min, and send the result to the display (User Channel 1 in **(E)**. **(B–F)** Identification of predefined motifs by the real-time closed loop procedure. In this example, the motif was defined as five spikes recorded sequentially from electrodes 37,26,25,34, and 41, at time intervals of at most 10 ms. Motif identification was followed by the generation of a trigger signal on digital out line 1 and by enforcing a refractory period of 1 sec during which no triggers were to be issued. **(B)** Spikes recorded from all electrodes over this 2 h period. Red triangles at bottom show times at which triggers were issued. **(C)** A portion of the recording shown at higher magnification. **(D)** Two examples of motifs identified in the segment shown in **(C)**. **(E)** A screenshot of CLEM's display during closed loop execution. Note the triggers issued on the digital out line and the output of the slow periodic closed-loop procedure to User Channel 1. **(F)** A screenshot showing how plugins are loaded and how arguments can be passed to them interactively. The ability to load and unload plugins allows the plugin to be modified without terminating CLEM. **(G)** The distribution of delays from last spike in the motif to the trigger. Note that delays were distributed more or less evenly across the duration of one closed-loop sample-analyze-output loop (~4 ms at a sampling rate of 16 kHz; see Figure 7A).

A 2 h session is shown in Figure 6. In this session, 78 motifs as defined above were identified (Figures 6B–E) with a delay of 9.8 ±1.1 ms (range 7.9–11.7; Figure 6G). Most of this delay stems from the fact that spikes are placed into the data repository only after their full waveforms are captured [Figure 1B (7, 8)] which in these runs was set to 8 ms (waveform length can be reduced if so desired), suggesting that reaction time stood consistently at a 0–4 ms. It is worth noting that in all these runs, computer CPU usage (eight cores) did not exceed 3–4% (including display, spike detection, storage and all other background processes).

Source code for this DLL as well as a second DLL for real time network burst detection (Eytan and Marom, 2006) can be found at https://github.com/Hananel-Hazan/CLEM-Plugin-Template.

### System performance

**Figure 7:**
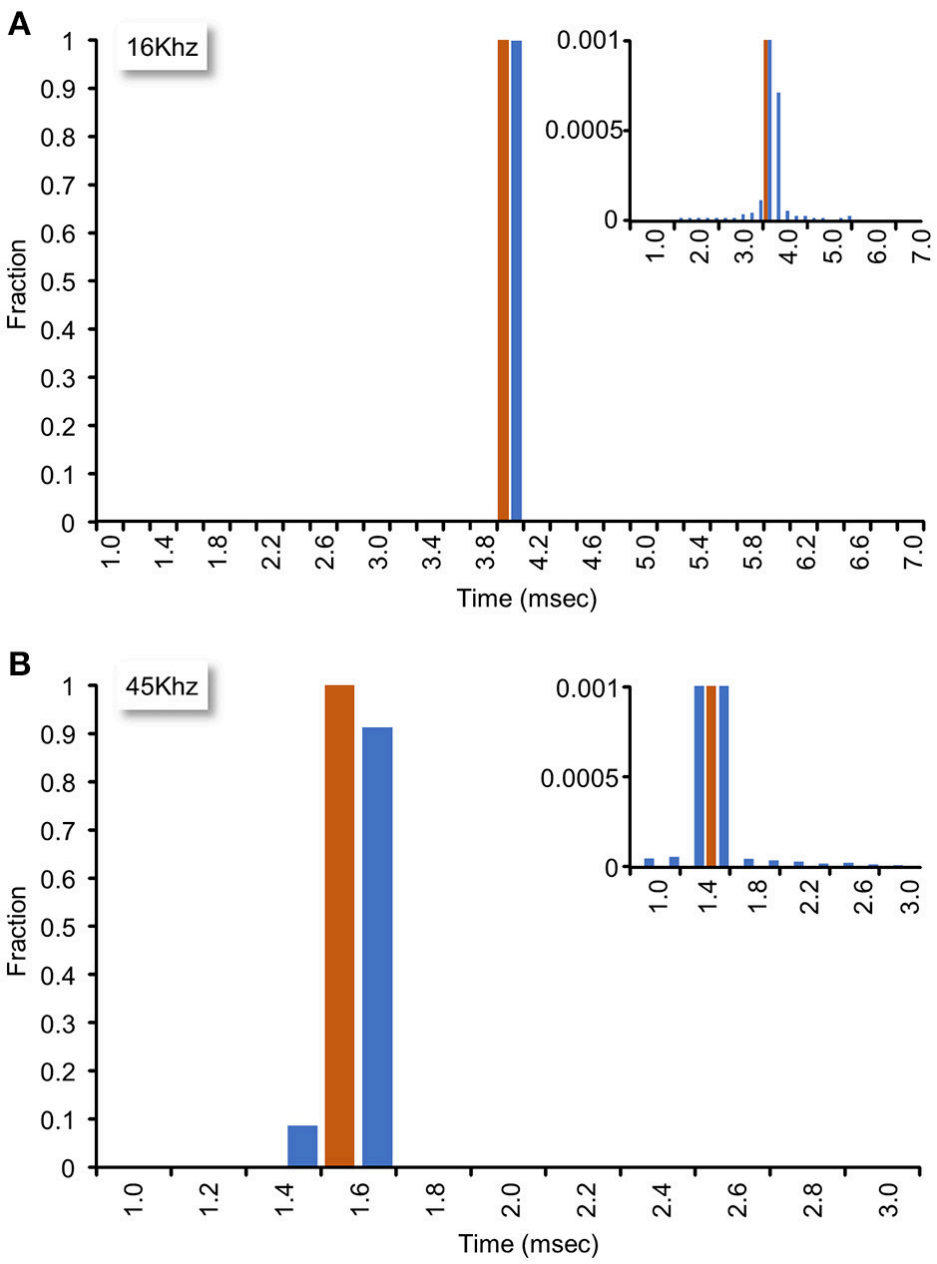
Closed loop performance. System performance was tested as explained in main text. **(A)** Sampling rate set to 16 kHz. Mean interval time was 3.94 ms (102,085 loops/iteration, 6 min run). Inset: Bottom of histogram at high magnification. **(B)** Sampling rate set to 45 kHz. Mean interval time was 1.40 ms (259,952 loops/iteration, 5.7 min run). Inset: Bottom of histogram at high magnification.

The system described here uses interrupts generated by the data acquisition card to clock sample-analyze-output loops. To measure the sample-analyze-output execution times this approach allows, and, importantly, the jitter in such times, we wrote a small plugin in which the real-time closed loop procedure (1) recorded time intervals (in sample units) between consecutive calls (“timestamp based measurements”), and (2) changed an output signal based on the input signal, and then measured the time (in analog to digital sample units) since the command was issued until an actual change was physically detected in a subsequent iteration (“I/O based measurements”). For the latter tests, we connected an Analog-out channel to one of the 64 Analog-in channels. During each iteration, the real-time closed loop procedure examined the value of the input channel, and set the analog output accordingly: For input values <1 V, the output was set to 5 V; conversely, for input values >1 V the output was set to 0 V. Because the minimal time interval between consecutive interrupts was determined by the time needed to collect the smallest allowable number of samples (64 samples for each of the 64 input channels), sample-analyze-output loop time intervals depended on sampling frequency (i.e., higher sampling frequencies resulted in tighter loops). We thus tested system performance at two sampling frequencies (16 and 45 Khz). As shown in Figure 7, mean interval times were 3.94 and 1.40 ms at 16 and 45 Khz, respectively (102,085 and 259,952 loops/iteration, ~6 min runs) with almost negligible jitter (Figure 7, insets). These data thus suggest that the system can perform sample-analyze-output loops at repetition rates exceeding 700 Hz in a very reliable fashion.

## Conclusion

During recent years, multiple closed loop platforms for neurophysiological studies have been put forward. It might thus be asked why yet another platform is necessary? Indeed, many excellent platforms are now available, with the performance of some of them exceeding that of CLEM. It should be noted, however, that many of such platforms require specialized hardware (such as CMOS ASICs, SOCs, FPGAs) some of which is not commercially available. Where commercial systems are concerned, dedicated hardware (as well as software) tends to be very costly in comparison to the system described here. Others systems based on widely available software (e.g., Matlab) and inexpensive hardware, (e.g., generic PCs serving as real time targets) are useful but somewhat ungainly, as they are based on multiple computers, require hardware tweaks, are remarkably sensitive to software versions and releases, and often lack informative and intuitive user interfaces. Finally, our own experience with such systems has taught us that the latter point is crucially important—we find that real time feedback, in the form of data visualization, as well as the ease to set up and document experiments is paramount. Moreover, we find that isolating the experimenter from standard tasks (data visualization, storage, etc.), exposing only powerful and flexible interfaces greatly accelerates progress while reducing mistakes during development and experimentation. Yet, as the platform is open and its source code in the public domain, experimenters willing to put in the required effort can further improve it and adapt it to changing needs. Moreover, our insistence to adhere to a mainstream OS and generic hardware should guarantee a reasonably long life cycle and justify the time investment and commitment associated with the selection of any system. Finally we wish to stress that although CLEM was developed using cultured neurons growing on MEA dishes, the system can be used in any experimental setting where neuronal waveforms are recorded from up to 64 channels. All that is required is a physical connection of such channels to the data acquisition board used here, which is readily realized using a commercial break-out box or screw terminal. We thus hope that CLEM will find a place in the gallery of open platforms, and prove to be useful to anyone in need of an easy to use, inexpensive, yet powerful multichannel electrophysiology platform with flexible closed loop abilities.

## Materials and methods

### Cell culture

Primary cultures of rat neurons were prepared according to a protocol approved by the “Technion, Israel Institute of Technology Committee for the Supervision of Animal Experiments” (ethics approval number IL-019-01-13). Briefly, cortices of 1–2 days-old Wistar rats of either sex were dissected, dissociated by trypsin treatment followed by trituration using a siliconized Pasteur pipette. 0.8–1.1 × 10^6^ cells were plated onto thin-glass MEA dishes (Multichannel Systems) containing 30 μm diameter electrodes arranged in an 8 × 8 array. MEA dishes were pre-treated with polyethylenimine (PEI, Sigma) to facilitate cell adherence. Preparations were kept in a humidified tissue culture incubator and grown in medium containing minimal essential medium (MEM, Sigma), 25 mg/l insulin (Sigma), 20 mM glucose (Sigma), 2 mM L-glutamine (Sigma), 5 mg/ml gentamycin sulfate (Sigma) and 10% NuSerum (Becton Dickinson Labware). Half of the volume was replaced three times a week with feeding medium similar to the medium described above but devoid of NuSerum, containing a lower L-glutamine concentration (0.5 mM) and 2% B27 supplement (Invitrogen).

### Multielectrode setup

The setup used for collecting data shown in Figures 1, 6 was based on the system described in Table 2 (Rig 2) running CLEM and a 60-channel MEA amplifier (MEA1060-BC; MCS). The 60 channels of amplified and filtered data were connected to 60 of the 64 analog to digital input channels of the PD2-MF-64-3M/12 data acquisition board using a home built connection box. This box also provided connections to the board's analog out channels as well as four digital output lines. To maintain neuronal network viability during recordings, the MEA dishes were heated to 37°C using the amplifiers built in heating base and a commercial controller (Multichannel Systems), and covered with a custom built “cap” equipped with ports through which a sterile air mixture was streamed into the dish, and perfusion media introduced and removed. In addition, the cap contained a dipping reference electrode made of thin platinum. The preparations were continuously perfused with feeding media at a rate of 2 ml/day using silicone tubes connected to the cap through the aforementioned ports, and an ultra-slow peristaltic pump (Instech Laboratories Inc., USA). In addition, a 95% air/5% CO_2_ sterile mixture was streamed continuously into the dish at rates regulated by a high precision flow meter (Gilmont Instruments, USA).

## Author contributions

HH coded the software, analyzed its performance and wrote the manuscript. NEZ conceived the project, coded the software, and wrote the manuscript.

### Conflict of interest statement

The authors declare that the research was conducted in the absence of any commercial or financial relationships that could be construed as a potential conflict of interest.
