# Supplementary material for: Closed Loop Experiment Manager (CLEM)—An Open and Inexpensive Solution for Multichannel Electrophysiological Recordings and Closed Loop Experiments
Source: Front Neurosci. 2017 Oct 18;11:579. doi: 10.3389/fnins.2017.00579 (PMC5651259; doi:10.3389/fnins.2017.00579)
Supplement: Supplementary file 1 [file DataSheet1.ZIP › Code Reference.docx]

# Hannanel Hazan, Noam E. Ziv

# Closed Loop Experiment Manager (CLEM) – A Simple and Inexpensive System for Multichannel, Closed Loop Electrophysiological Experimentation

# Supplementary Text 1

# Writing Plugins for CLEM - Reference

This document provides a reference for writing Plugins for CLEM

As described in the main text, writing an effective plugin entails fleshing out five functions described below (DLL_Init, DLL_Engage, DLL_Disengage, RealTime_CL and SlowPeriodic_CL ) in the form of a MS Windows DLL. These functions have access to the global data repositories through a data structure described later, which contains pointers (64bit) to all major items in this repository, as well as additional system variables that can be accessed if needed. The data structure is defined in the file GlobalVar.h.

Note that it is not mandatory to flesh out all functions.

## **Functions**

***1) DLL_Init***

void DLL_Init (CoreFunc_Global_var *corefunc_global_var, char *str)

Arguments:

corefunc_global_var a pointer to a *CoreFunc_Global_var* data structure defined in GlobalVar.h.

str a pointer to an array of 8 bit characters which can be used to output text to the GUI Text message window. Maximal allowed string length is 200 characters.

Description

This function is called only once, when the DLL is loaded. The purpose of this function is to provide the plugin with a pointer to a data structure that provides access to global data repositories and associated variables.

It is recommended to copy this pointer to a DLL-wide global copy of this pointer for subsequent use by all DLL functions, for example

Global_Var = corefunc_global_var;

This function can also be used to load configuration data, initialize internal variables, test external devices, etc.

If the user wishes to use the slow periodic closed loop pipeline, the frequency at which the slow periodic close loop function will be called can be specified by assigning a value to the field DLL_Slow_Process_Running_Freq, (in repeats/sec), for example

Global_Var->DLL_Slow_Process_Running_Freq = 2.0;

The function can send a text message to the GUI text message window by copying a null terminated character string to *str*. Note the limit of 200 characters, including the terminating null character.

***2) DLL_Engage***

void

DLL_Engage(int argc, char **argv, char *str)

Arguments:

argc integer [32bit] – the number of arguments passed to the function.

argv a pointer to an array of pointers, each of which points to an argument string (arrays of 8bit characters).

str a pointer to an array of 8 bit characters which can be used to output text to the GUI Text message window. Maximal allowed string length is 200 characters.

Description

This function is called each time the user presses the “Engage” button on the GUI. The function is called just before the functions RealTime_CL and SlowPeriodic_CL are called for the first time. Can be used to set up the close loop runs by initializing counters, opening files, etc.

When engaging closed loop plugins, the user has the option to provide arguments through the GUI. Arguments can be comma (,) Tab or Space delimited. CLEM parses the argument string into separate strings, passes the number of arguments through *argc* variable and passes the argument strings themselves through *argv*.

The function can send a text message to the GUI text message window by copying a null terminated character string to *str*. Note the limit of 200 characters, including the terminating null character.

***3) DLL_Disengage***

void

DLL_Disengage(char *str)

Arguments:

str a pointer to an array of 8 bit characters which can be used to output text to the GUI Text message window. Maximal allowed string length is 200 characters.

Description

This function is called when user presses the “Disengage” button on the GUI. The function is called just after the last time the functions RealTime_CL and SlowPeriodic_CL are called. The purpose of this function is to execute tasks such as summarizing data collected during a closed loop session, closing files etc.

The function can send a text message to the GUI text message window by copying a null terminated character string to *str*. Note the limit of 200 characters, including the terminating null character.

***4) RealTime_CL***

void

RealTime_CL (double *A_out, float *U_out, unsigned short *D_Out, char *str)

Arguments:

A_out Pointer to a variable used to send data to Analog Out hardware channels (currently 2 channels). To output data, copy an array (currently 2 elements, one for each channel) of values (of type *double*) to the memory area pointed to by this pointer. The actual number of analog out channels can be found in

*Global_Var->Analog_Output_Channel*.

U_out Pointer to a variable used to send user data (2 separate channels) to the display and permanent storage. To send this data, copy an array (currently 2 elements, one for each channel) of values (of type *float*) to the memory area pointed to by this pointer. The actual number of user data channels can be found in

*Global_Var->* *Number_of_User_Data_Channels*.

D_Out Pointer to a variable used to send digital out signals. To send data to digital outputs, copy an 8 bit value to the memory area pointed to by this pointer.

str A pointer to an array of 8 bit characters which can be used to output text to the GUI Text message window. Maximal allowed string length is 200 characters.

Description

This function executes the user-defined, real-time closed loop tasks. This is where the actual tasks to be done should be coded. Note that the time available for task execution is inversely related to the frequency at which the function is called. This, in turn depends on the sampling frequency of the DAQ card. For example, if sampling rate is 16 KHz, the function will be called every ~4 ms. Consequently, tasks performed in each iteration should be limited to this time frame, while lengthy calculations, as well as time consuming IO activities should be avoided. The function can generate output that will be directed to analog and digital outputs, generate user data that will be displayed by the GUI and stored to permanent storage devices if desired and send text messages to the Text message window.

***5) SlowPeriodic_CL***

void SlowPeriodic_CL (double *A_out, float *U_out, unsigned short *D_Out, char *str)

Arguments:

A_out Pointer to a variable used to send data to Analog Out hardware channels (currently 2 channels). To output data, copy an array (currently 2 elements, one for each channel) of values (of type *double*) to the memory area pointed to by this pointer. The actual number of analog out channels can be found in

*Global_Var->Analog_Output_Channel*.

U_out Pointer to a variable used to send user data (2 separate channels) to the display and permanent storage. To send this data, copy an array (currently 2 elements, one for each channel) of values (of type *float*) to the memory area pointed to by this pointer. The actual number of user data channels can be found in

*Global_Var->* *Number_of_User_Data_Channels*.

D_Out Pointer to a variable used to send digital out signals. To send data to digital outputs, copy an 8 bit value to the memory area pointed to by this pointer.

str A pointer to an array of 8 bit characters which can be used to output text to the GUI Text message window. Maximal allowed string length is 200 characters.

Description

This function executes slow, periodic closed loop tasks. This is where such tasks should be coded. The time available for task execution is inversely related to the frequency at which the function is called, which is defined in *DLL_Init* as described above. Tasks performed in each iteration can be as time consuming as necessary, as long as they are completed in the allotted time. The function can generate output that will be directed to analog and digital outputs, generate user data that will be displayed by the GUI and stored to permanent storage devices if desired and send text messages to the Text message window.

## Accessing CLEMs global data repositories

***Raw A/D values.***

CLEM stores raw potential measurements of the 64 analog in channels for a user-defined duration. These data can be accessed via the variable *Global_Var->RAW_Input_Volts* where *Global_Var* is a copy of the pointer to global variables provided as part of the *DLL_Init* function described above.  *RAW_Input_Volts* is a vector that contains the raw sample values (in volts, as 32bit floating-point values) of all channels. Its length equals the number of channels times the number of samples saved for each channel (a value stored in the global variable *Global_Var->Samples_per_Channel*).

To access the value of samples belonging to a particular channel the user can use the array *Global_Var-> Pointer_To_Electrode_In_ RAW_Input_Volts* that contains pointers to the first location of data of each channel in the vector *Global_Var->RAW_Input_Volts*. For example to access the 350^th^ sample for channel 12:

float value;

value = *(Global_Var->RAW_Input_Volts [Global_Var-> Pointer_To_Electrode_In_Memory_Input_Volt[12]] + 350);

Similarly, to access the most recent sample of each channel the variable *Global_Var-> Location_of_Last_Value_of_RAW_Analog_Input* can be used (the same value applies to all channels, as data is not interleaved but rather stored separately for each channel). As this variable points to the location to which the *next* sample will be placed, the most recent sample can be found by subtracting 1 from this value. For example to access the most recent sample of channel 17, the user can write:

float value;

value = *(Global_Var->RAW_Input_Volts [Global_Var-> Pointer_To_Electrode_In_Memory_Input_Volt[17]] + Location_of_Last_Value_of_RAW_Analog_Input - 1);

In practice, however, data is stored in round robin fashion. Thus, when the memory block allocated for the samples of each channel is full, the next samples are written to the beginning of the same block, overwriting the oldest samples.

Consequently, if one is interested to scan backward through stored samples in order to examine all of the most recent samples collected for a certain channel (17 in this example), the following code can be used:

__int64 i, n_samples, offset;

n_samples = Global_Var-> Samples_per_Channel;

offset = Global_Var-> Location_of_Last_Value_of_RAW_Analog_Input;

for (i = 0 ; i < n_samples ; i++)

{

offset--;

if (offset < 0)

offset = n_samples – 1;

value = *(Global_Var->RAW_Input_Volts [Global_Var-> Pointer_To_Electrode_In_Memory_Input_Volt[17]] + offset);

// do something with the sample

}

Every sample has a system *time stamp* associated with it. The time stamp is in sample units, which is reset to zero upon system initialization. Thus, given a sampling rate of 16kHz, a sample collected 30 seconds after system initialization would have the timestamp 30x16,000 = 480,000. As data is collected simultaneously for all channels, samples collected for all channels will have the same time stamp.

The time stamp of each sample is stored in the global variable *Global_Var->Time_Stamp_Counter_Array*. Thus the time stamp associated with the most recent samples can be found using the following code

__int64 offset, timestamp;

offset = Global_Var-> Location_of_Last_Value_of_RAW_Analog_Input - 1;

if (offset < 0)

offset = Global_Var-> Samples_per_Channel – 1;

timestamp = *(Global_Var->Time_Stamp_Counter_Array + offset);

If the time stamp for a sample at a particular index in *Global_Var->RAW_Input_Volts* is zero, it means that data has not yet been collected for this particular sample index in memory.

***Events***

Apart from raw A/D values, all other data in shared data repositories is stored as lists of events. Such events include action potentials (spikes), analog output, digital input and output, and user data. The length of all event lists is given by the variable *Global_Var->Size_Of_Events_In_Memory.* Each of these lists can be accessed using the following variables:

*Global_Var->****Analog_Spikes_Events***

A pointer to an array of data structures, each of which contain information on one spike detected by CLEM. Each data structure in this array contains the following fields:

- *timestamp:* (unsigned 64 bit integer) contains the time (in sample units as described above) at which the spike occurred.
- *channel:* (unsigned 16 bit integer) contain the channel number on which the spike was detected.
- *value:* (pointer to 32 bit float) an array of voltage values around the spike detection time (the spike envelope). The length of this array is a sum of the global variables *Global_Var-> Pre_Trigger_Samples* & *Global_Var->Post_Trigger_Samples*.

*Global_Var->****Analog_Output_Events***

A pointer to an array of data structures which contain information on changes to analog outputs. Each data structure in this array contains the following fields:

- *timestamp:* (unsigned 64 bit integer) contains the time (in sample units as described above) at which the change occurred.
- *channel:* (unsigned 16 bit integer) that contains the channel number of the analog output on which the change occurred.
- *value:* (pointer to an array of 32 bit float). The number of elements is defined by *Global_Var->*buffer_Output_Per_Channel

*Global_Var->***Digital_I_Events** and *Global_Var->***Digital_O_Events**

Both are pointers to arrays of data structures that contain the timestamp and values of digital input or output lines. Each data structure in this array contains the following fields:

- *timestamp:* (unsigned 64 bit integer) contains the time (in sample units as described above) at which the change occurred.
- *value:* (unsigned 16 bit integer) 8 bit value for all input or output lines

*Global_Var->***User_Analog_Events**

A pointer to an array of data structures which contain plugin-generated values (user data) that will be displayed as graphs in the GUI

- *timestamp:* (unsigned 64 bit integer) contains the time (in sample units as described above) at which the values were set.
- *value:*  (pointer to 32 bit float) A two element array that contains the user generated values.

**Accessing events**

Each event list is associated with a variable (an unsigned 64 bit integer) that points to position of the most recent entry within the array. These are

- *Global_Var->* ***Location_of_Last_Event_Analog_Spike***
- *Global_Var->****Location_of_Last_Event_Analog_Output***
- *Global_Var->****Location_of_Last_Event_Digital_I***
- *Global_Var->****Location_of_Last_Event_Digital_O***
- *Global_Var->****Location_of_Last_Event_User_Analog***

Here too, events are stored in round robin fashion. Thus, to walk backward through the list of events, wraparound has to be considered. Below is an example showing how one might count the number of spikes detected in all electrodes during the past 10 seconds:

__int64 timestamp_now, i, offset;

int count;

// get the timestamp of the most recent sample

offset = Global_Var-> Location_of_Last_Value_of_RAW_Analog_Input - 1;

if (offset < 0)

offset = Global_Var-> Samples_per_Channel – 1;

timestamp_now = *(Global_Var->Time_Stamp_Counter_Array + offset);

// calculate the equivalent of 10 seconds in sample units

delta_t_in_samples = 10 * Global_Var->Input_Hz;

// move back through the list and count spikes

i = Global_Var->Location_of_Last_Event_Analog_Spike;

count = 0;

while ((timestamp_now - Global_Var->Analog_Spikes_Events[i].timestamp) > delta_t_in_samples)

{

count++;

i--;

if (i < 0)

i = Global_Var->Size_Of_Events_In_Memory - 1;

}

**Additional variables in GlobalVars**

The data structure to which a pointer is provided to in DLL_init contains several additional useful variables described in more detail in the file GlobalVars.h

It is important to understand that these variables are used by all of CLEMs threads; therefore, it is up to the user writing the plugin to avoid data corruption caused by inadvertently changing these variables values.
